# Region-Specific Long-Term Transcriptional Changes in the Plasminogen Activation System and Neuroinflammation in the Rat Brain After Status Epilepticus: Association with Depressive-like Behavior

**DOI:** 10.3390/brainsci15101083

**Published:** 2025-10-07

**Authors:** Anna Karan, Elizaveta Selivanova, Yulia Spivak, Elena Suleymanova

**Affiliations:** Department of Molecular Neurobiology, Institute of Higher Nervous Activity and Neurophysiology, Russian Academy of Sciences, Butlerova 5A, Moscow 117485, Russia; akartar.n@gmail.com (A.K.); solntseva.yelizaveta@mail.ru (E.S.); lampo_love@mail.ru (Y.S.)

**Keywords:** epilepsy, status epilepticus, plasminogen activation system, cytokines, growth factors, depression

## Abstract

**Background/Objectives**: Growing evidence implicates that processes mediated by cytokines, growth factors, and the plasminogen activation (PA) system play crucial roles in the pathogenesis of epilepsy and its comorbidities. **Methods**: This study was carried out on the lithium–pilocarpine rat model of status epilepticus (SE). We investigated mRNA expression patterns of PA system components (tPA/PAI-1/uPAR), pro-inflammatory cytokines (IL-1β/TNF-α), and TGF-β1 in the hippocampus and cortex 7 days (latent period) and 5 months (chronic period) after SE. In the chronic period, rats were subjected to the sucrose preference test for the evaluation of depressive-like behavior. **Results**: Our results revealed region-specific dysregulation of the PA system that persisted into the chronic period, with tPA (*Plat*) transiently upregulated in the dorsal hippocampus during the latent phase while uPAR (*Plaur*) exhibited sustained elevation in the entorhinal cortex into the chronic period. TGF-β1 (*Tgfb1*) exhibited widespread upregulation across all examined brain regions during the latent period, remaining elevated in the ventral hippocampus 5 months after SE. Notably, latent-phase neuroinflammation showed cortical specificity, with IL-1β (*Il1b*) expression increased in the frontal cortex while the hippocampal expression remained unchanged. The subgroup of rats displaying anhedonia (reduced sucrose preference) after SE exhibited higher *Tgfb1* and *Tnf* expression in the ventral hippocampus and entorhinal cortex compared to non-anhedonic subgroup of rats and the control group (no SE) in the chronic period. **Conclusions**: Our findings demonstrate persistent, region-specific transcriptional changes in the PA system following SE, with higher expression of *Tgfb1* and *Tnf* in a subgroup of rats with more severe functional outcome in the chronic period after SE.

## 1. Introduction

The role of cytokines and growth factors mediating neuroinflammation, neurodegeneration, neuronal plasticity, and other processes in the pathogenesis of various brain disorders has been extensively studied. Epilepsy is one of the most widespread neurological disorders, affecting around 50 million people worldwide and diagnosed in 5 million people each year according to WHO. Despite multiple studies of the pathogenesis of epilepsy, many aspects of the mechanisms leading the development of seizures and various comorbidities of epilepsy remain unclear.

The growing body of evidence suggests that the plasminogen activation (PA) system plays a significant role in a number of neurologic disorders, including epilepsy [1]. The PA system refers to a group of proteins, which includes plasminogen activators, their inhibitors, and receptors, involved in the conversion of plasminogen into serine protease plasmin and plays a key role in fibrinolysis [2]. In the central nervous system, it is involved in the regulation of a variety of processes such as cell growth and migration, neuronal plasticity, blood–brain barrier permeability, and microglia activation [3,4]. Various impacts on the brain, including trauma [5], ischemic damage [6], and seizures [7,8], are known to induce the expression of the components of the PA system. Such upregulation has been shown in areas of inflammation [9], thus being involved in the processes related to neuroinflammation. Several studies have shown upregulation of PA system components in response to seizure activity [7,10]. An increase in the activators of plasminogen mRNA expression was reported in status epilepticus (SE) models, and the acute increase in the expression of tissue activator of plasminogen (tPA) persisted in the chronic period after SE [11,12,13]. An increase in PA system components expression was also found in human focal epileptogenic pathologies [1].

As noted above, the upregulation of the PA system in response to seizures is closely intertwined with the processes of neuroinflammation. A large body of data has been accumulated about the critical role of neuroinflammation and inflammatory cytokines in seizures and epileptogenesis [14]. In symptomatic epilepsy, the development of chronic seizures occurs after an initial insult, damaging the brain tissues and triggering a cascade of processes, including changes in the expression of a number of cytokines and growth factors. Interleukin-1β (IL-1β) and tumor necrosis factor α (TNF-α) are important pro-inflammatory cytokines, which are considered to be the among the main cytokines involved in the pathogenesis of epilepsy [15]. Elevated levels of IL-1β were reported in epilepsy patients and in experimental models of epilepsy [14], and the majority of studies suggest that elevated IL-1β levels increase seizure susceptibility and promote epileptogenesis [15]. A TNF-α increase in limbic structures in response to seizure activity was found in animal models [16,17], and in some studies, elevated levels of TNF-α in epilepsy patients were associated with higher severity of the disease [18].

TGF-β1, a member of TGF-β family of growth factors, is a multifunctional cytokine involved in a number of processes in the central nervous system under the normal conditions and pathology, including intracellular matrix regulation, cell growth and differentiation, and immune response [19]. TGF-β signaling pathways are complex, and TGF-β function depends on the context [20]. In the neuroinflammatory response, TGF-β1 acts as a cytokine with both pro- and anti-inflammatory properties [20]. TGF-β signaling is reported to promote the development of anti-inflammatory microglia phenotype [21,22], but it was shown that it can activate pro-inflammatory cytokine expression by astrocytes [23].

Rapid upregulation of TGF-β1 in response to various types of brain damage was reported [24]; however, the functional role of TGF-β1 in the development of pathologic alterations apparently can differ depending on the type of injury and the context. In the traumatic brain injury model, rats with TGF-β1 knockdown demonstrated aggravation of neurologic deficit after the trauma [25], while TGF-β1 agonists attenuated inflammatory response and reduced cognitive deficit in ischemic demyelination model [26]. In the experimental ischemic stroke, multiple studies reported the protective role of TGF-β1 [27,28,29,30]. Treatment with exogenic TGF-β1 was reported to reduce neuronal death and improve cognitive function in the lithium–pilocarpine model of SE in rats [31].

Recent studies, however, show that TGF-β1 could play a more complex role in neurological disorders. Despite its reported anti-inflammatory and neuroprotective effects, TGF-β1 has been shown to promote neuronal hyperexcitability and contribute to epileptogenesis [32]. There are reports of an association between increased TGF-β1 levels and drug-resistant epilepsy in patients [33], and a number of studies on experimental models have reported the involvement of TGF-β1 in epileptogenesis [20,23,34,35].

Interestingly, there is evidence that pro-inflammatory cytokines [36], the components of PA system [37], and TGF-β1 [38] play important roles in the pathogenesis of behavioral disorders, including depression. Increased blood levels of TGF-β1 have been reported in patients with depressive disorders [39,40], and the involvement of TGF-β1 in the development of depressive-like behavior has been demonstrated in animal models [41,42]. At the same time, epilepsy is often associated with comorbid psychiatric disorders with prevalence of depression [43]. Neuroinflammation-related abnormalities could be a substrate of comorbidity between epilepsy and psychiatric disorders, including depression [44].

Animal models of epilepsy allow us to investigate alterations in the expression of various proteins at different stages of the pathogenesis of epilepsy. Lithium–pilocarpine model reproduces many features of human temporal lobe epilepsy (TLE) [45,46]. In this model, status epilepticus (SE) acts as a damaging impact triggering epileptogenesis [47]. This model is characterized by the presence of seizure-free latent period lasting from a few days to a few weeks, when a cascade of pathologic processes, including activation of inflammatory pathways, takes place [48]. The lithium–pilocarpine model is characterized by the development of behavioral changes reproducing some features of neuropsychiatric comorbidities in epilepsy patients. It was demonstrated that SE led to the development of depressive-like behavioral impairments, which included the lack of preference of sweet taste–anhedonia [49,50].

In this study, we investigated changes in the mRNA expression of the PA system components, including tissue plasminogen activator (tPA), its inhibitor PAI-1, and urokinase-type plasminogen activator receptor (uPAR) in the latent and chronic periods after SE in rats. Changes in mRNA expression were studied in the limbic structures (the hippocampus and the entorhinal cortex), which are among the most severely affected regions during SE [51], as well as in the neocortical regions (somatosensory and frontal cortex). The neuronal damage induced by SE is not restricted to the limbic structures and can also affect the neocortex [52], but these regions can exhibit different patterns of inflammatory response to seizures [53]. We also examined the relationship between the PA system components and pro-inflammatory cytokines and TGF-β1, as well as the relationship between these cytokines’ expression and the development of depressive-like behavior in rats in the chronic period after SE. 

## 2. Materials and Methods

### 2.1. Animals

Adult male Wistar rats weighing 200–250 g at the beginning of the experiments were used in the study. All animals (*n* = 74) were obtained from the Scientific Center for Biomedical Technologies of the Federal Medical and Biological Agency, Russia. The rats were kept under standard vivarium conditions with a 24 h light/dark cycle and free access to food and water. All experimental procedures were conducted in accordance with Directive 2010/63/EU for animal experiments and were approved by the Ethics Committee of the Institute of Higher Nervous Activity and Neurophysiology of the Russian Academy of Sciences (protocol no. 8, 28 October 2024). The animals were allowed to acclimatize in the vivarium for no less than two weeks and then randomly distributed between the control and experimental groups.

### 2.2. Lithium–Pilocarpine Model of SE

Seizure activity was induced in rats by administration of 127 mg/kg lithium chloride (Acros Organics, Trenton, NJ, USA) and 30 mg/kg pilocarpine hydrochloride (Sigma, St. Louis, MO, USA) 24 h after lithium chloride injection. Seizures were observed for 90 min and scored according to the Racine scale [54]. To stop SE, rats were injected with 10% paraldehyde, 0.6 mL/kg (Sigma, St. Louis, MO, USA) dissolved in saline. Paraldehyde in this dose effectively suppresses convulsive activity and significantly increases the survival rate of animals after SE [55].

The control rats received saline instead of pilocarpine and did not develop seizures. All drugs were injected intraperitoneally. To improve the survival of the animals, they received 5% glucose solution orally for the next 2–3 days and were manually fed with wet food if significant weight loss was observed.

Only rats developing convulsive SE with seizures scored no less than 3–4 according to Racine scale were included in the study. The final sample sizes were determined according to the results of our previous experiments and the calculations carried out for *Serpine1* expression in the hippocampus using Altman’s nomogram with power of 0.8.

The following groups were included in the analysis: 7 days post-SE (*n* = 8), 7 days control (*n* = 9), 5 months post-SE (*n* = 13), 5 months age-matched control (*n* = 12).

### 2.3. Video Monitoring

Five months after SE, rats were video monitored to detect motor seizures. For this purpose, digital cameras were installed above the rat cages. During the dark hours, recordings were carried out under dim red light. Animals were video monitored for 24 h per day for 7 consecutive days. Video recordings were analyzed manually; clonic and tonic–clonic seizures were detected visually and scored according to the Racine scale.

### 2.4. Sucrose Preference Test

After the video monitoring was completed, rats were placed into individual boxes supplied with two identical bottles. On day 1 (pre-test), the bottles were filled with tap water and offered to the rats for the adaptation and measurement of water consumption. On days 2 (test 1) and 3 (test 2), one of the bottles was filled with 1% sucrose solution, and the other with tap water; the initial volume of each solution was 100 mL. After each 24 h period, the remaining volume of the liquid was measured. To avoid side preference, the bottles with water and sucrose were swapped once a day. Animals had unlimited access to food throughout the experiment. The total consumed volume of water and 1% sucrose per 48 h was measured and the percentage of sucrose consumption was calculated as a % of the total consumed liquid volume.

### 2.5. qPCR

To carry out qPCR, the following brain structures were extracted and immediately frozen in liquid nitrogen: dorsal and ventral hippocampus, entorhinal cortex, somatosensory cortex, and frontal cortex. Samples were gathered 7 days and 5 months after SE.

Total RNA was extracted using ExtractRNA reagent (Evrogen, Moscow, Russia). To eliminate genomic DNA contamination, RNA samples were treated with DNase I (Thermo Scientific, Waltham, MA, USA). MMLV RT reagent kit (Evrogen, Moscow, Russia) and murine RNase Inhibitor (New England Bio-labs, Ipswich, MA, USA) were used to carry out reverse transcription. An equimolar mixture of random decaprimer (Evrogen, Moscow, Russia) and Oligo(dT)15 primer (Evrogen, Moscow, Russia) was used; with each primer at a final concentration of 1 μM. After reverse transcription, the reaction mixture was diluted eightfold with deionized water. The mRNA expression of the following genes was analyzed: *Serpine1* (PAI-1), *Plat* (tPA), *Plaur* (uPAR), *Il1b* (IL-1β), *Tnf* (TNF-α), and *Tgfb1* (TGF-β1). Relative quantities of mRNAs for the genes of interest were measured in a Bio-RadCFX-384 real-time PCR station using a qPCRmix-HS SYBR + LowROX PCR mix for PCR (Evrogen, Moscow, Russia) according to the manufacturer’s protocol. Relative quantities of mRNAs of target genes were normalized to the geometric mean of the mRNA expression levels for the *Ywhaz*, *Osbp*, and *Hprt1* genes [56]. The stability of reference genes was evaluated using geNorm and NormFinder analysis. A negative control with the product of DNase I treatment was run for all the samples and genes to assess the quality of the DNase treatment. Gene expression was analyzed by the E^−∆∆Ct^ method. The results of qPCR were represented as relative expression. The sequences of used primers and amplicon sizes are shown in Table 1.

### 2.6. Statistical Analysis

The statistical analysis of the qPCR results was carried out using programs written in the R language (version 4.2.2) and Statistica software 12.0 (StatSoft, Tulsa, OK, USA). The data were preliminarily checked for normality using the Shapiro–Wilk test, and it showed that most data samples did not have normal distribution. Therefore, we used the nonparametric Mann–Whitney U test for comparing experimental groups and the Kruskal–Wallis test for multiple comparisons with Mann–Whitney’s post hoc test. The nonparametric Spearman test was used for the evaluation of correlations between gene relative expression values (fold expression). To reduce the effect of multiple comparisons across different genes and structures, false discovery rate (FDR) analysis via the Benjamini–Hochberg procedure was carried out.

The data were presented as the mean ± standard error of the mean (SEM). The level of significance was set at *p* < 0.05.

## 3. Results

### 3.1. Lithium–Pilocarpine Seizures

Pilocarpine injection induced the development of severe convulsive seizures in rats. The onset of Racine stage 4 motor seizures was considered as SE start, and only rats developing persistent stage 3 and stage 4 seizures were included in the study. After the administration of paraldehyde, rats remained sedated for 4–6 h; after that, no recovery of motor seizure activity was observed.

The limitations of the present study include the low confirmed incidence of motor spontaneous seizures in the chronic period after SE. Out of 13 rats observed 5 months after SE, only 3 rats were confirmed to demonstrate spontaneous motor seizure activity. Therefore, we have not analyzed the effects of spontaneous seizures in the chronic group.

### 3.2. Expression of Serpine1, Plat, and Plaur in the Hippocampus, Entorhinal Cortex, and Neocortex

**Figure 1:**
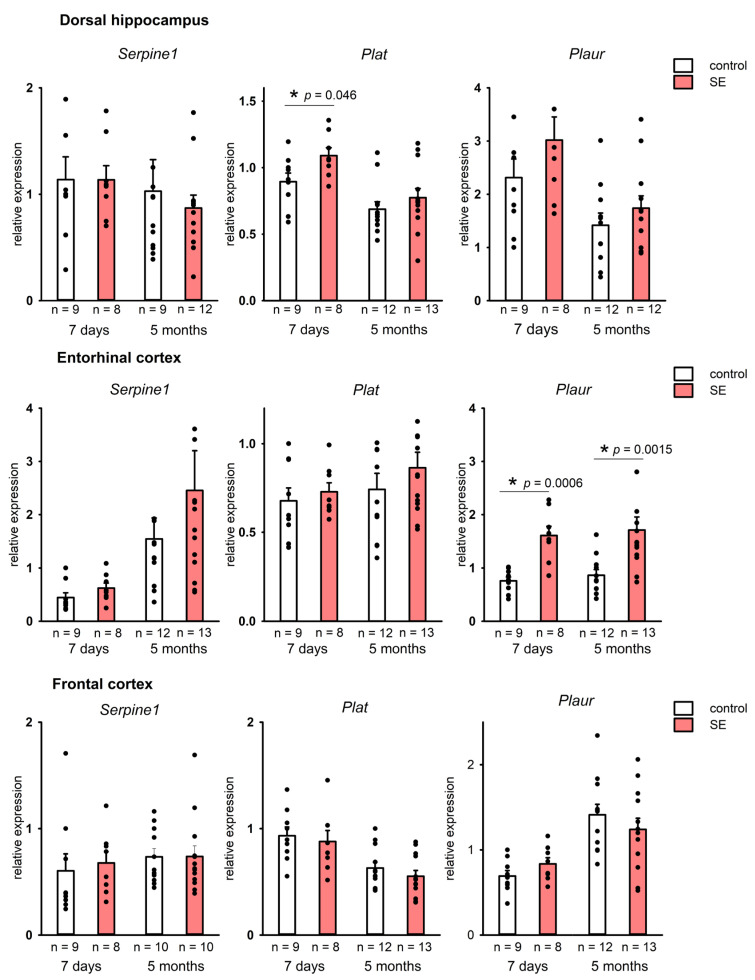
Expression of *Serpine1, Plat*, and *Plaur* in the dorsal hippocampus and the entorhinal cortex 7 days and 5 months after SE. Control—age-matched control group; SE—post SE group; *n*—the number of samples in each group *—statistically significant difference at *p* < 0.05; raw *p*-values are shown, Mann–Whitney test. Data is presented as mean ± SEM.

The analysis of mRNA expression of Serpine1 (PAI-1), Plat (tPA), and Plaur (uPAR) genes encoding in the rat hippocampus showed a tendency toward upregulation of Plat in the dorsal hippocampus in the 7 days after SE (*p* = 0.046, MU test, FDR-adjusted *p* = 0.174). No upregulation was found in the chronic period after SE (5 months) (Figure 1). At the same time, we have found no statistically significant changes in the expression of Serpine1 and Plaur in either dorsal or ventral hippocampus 7 days after SE, and it remained unchanged 5 months after SE.

In the entorhinal cortex, on the contrary, a significant upregulation of Plaur (*p* = 0.00057, MU test, FDR-adjusted *p* = 0.0057) was observed 7 days after SE. A significant upregulation of Plaur was still detected in the entorhinal cortex 5 months post-SE (*p* = 0.0015, MU test, FDR adjusted *p* = 0.046) (Figure 1), whereas changes in Serpine1 and Plat expression were not found.

Neocortical regions did not show significant changes in the expression of Serpine1, Plat, and Plaur in either latent or chronic periods after SE (Figure 1). Therefore, a significant upregulation of plasminogen activation system components was observed in the limbic structures, including the hippocampus and the entorhinal cortex, but was not found in the neocortical regions.

**Figure 2:**
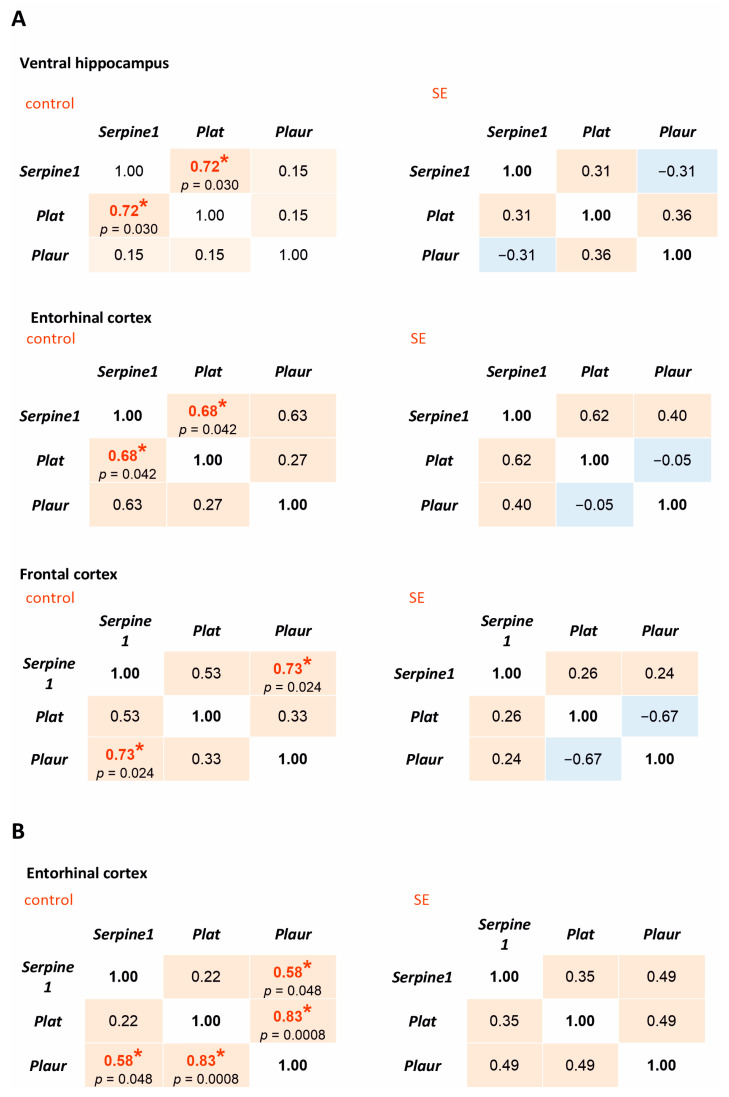
Spearman correlation coefficients for *Serpine1*, *Plat*, and *Plaur* expression in the rat hippocampus and cortex 7 days after SE (**A**) and 5 months after SE (**B**). Control—age-matched control group; SE—post SE group. Red fills indicate positive correlations, blue—negative correlations. *—statistically significant correlations at *p* < 0.05.

Correlation analysis of the expression of *Serpine1*, *Plat*, and *Plaur* showed significant positive correlations in the ventral hippocampus, entorhinal cortex, and the frontal cortex of the control rats. However, no statistically significant correlations were found in the latent period post-SE (Figure 2A). In the chronic period, significant positive correlations between *Serpine1*, *Plat*, and *Plaur* were observed in the entorhinal cortex of the control rats but not in the rats that survived 5 months after SE (Figure 2B). Therefore, even in the absence of statistically significant changes in the expression of *Serpine1*, *Plat*, and *Plaur*, the long-term disruption of mutual correlations between the components of plasminogen activation system at mRNA level occurred as a result of SE.

### 3.3. Expression of Il1b, Tnf, and Tgfb1 in the Hippocampus, Entorhinal Cortex, and Neocortex

In addition to the plasminogen activation system, we investigated the mRNA expression of pro-inflammatory cytokines IL-1β and TNF-α and anti-inflammatory factor TGF-β1 in the rat hippocampus and cortex.

**Figure 3:**
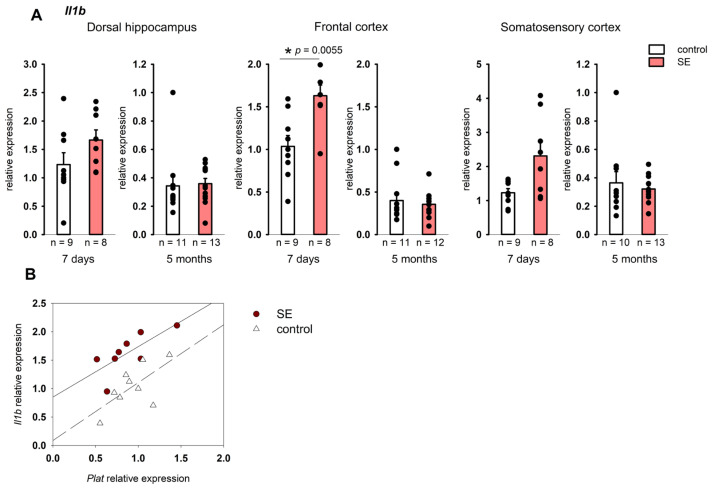
(**A**) Expression of *Il1b* in the cortex 7 days and 5 months after SE. Control—age-matched control group; SE—post SE group. *—statistically significant difference at *p* < 0.05; *n*—the number of samples in each group; raw *p*-values are shown, according to the results of the Mann–Whitney test. Data is presented as mean ± SEM. (**B**) correlations between *Plat* and *Il1b* expression in the frontal cortex of rats 7 days after SE (Spearman coefficient 0.74, *p* = 0.036) and control rats (Spearman coefficient 0.57, *p* = 0.111).

**Figure 4:**
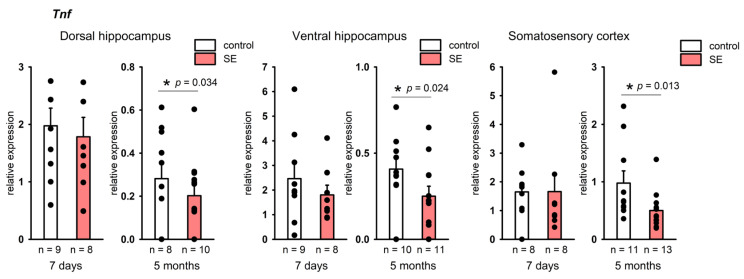
Expression of *Tnf* in the hippocampus and cortex 7 days and 5 months after SE. Control—age-matched control group; SE—post SE group. *—statistically significant difference at *p* < 0.05, *n*—the number of samples in each group; raw *p*-values are shown, according to the results of the Mann–Whitney test. Data is presented as mean ± SEM.

No changes in the expression of *Il1b* and *Tnf* were found in the hippocampus 7 days after SE. At the same time, a statistically significant increase in *Il1b* expression was found in the frontal cortex (*p* = 0.005, Mann–Whitney test, FDR-adjusted *p* = 0.027), and in the somatosensory cortex it tended to increase (*p* = 0.059, Mann–Whitney test), Figure 3A. In the chronic period, no increase in *Il1b* expression was found in either the hippocampus or the cortex, while *Tnf* was downregulated in the dorsal and ventral hippocampus and the somatosensory cortex (*p* = 0.034, *p* = 0.024, and *p* = 0.013, respectively, according to the Mann–Whitney test), Figure 4. However, in the chronic period, the results were characterized by high individual variability, and FDR adjustment resulted in *p* = 0.19, *p* = 0.21, and *p* = 0.21, respectively.

The analysis of the relationship between the expression of pro-inflammatory cytokines and the components of plasminogen activation system showed statistically significant correlations of *Il1b* expression with *Plat* in the frontal cortex of post SE rats 7 days after SE (Spearman coefficient 0.74, *p* = 0.036), while in the control group the correlation was not significant (Figure 2B). Unlike *Il1b*, *Tnf* expression did not correlate with the expression of plasminogen activation system components either 7 days or 5 months after SE.

Our results showed a significant upregulation of *Tgfb1* across the studied structures: in the dorsal and ventral hippocampus (*p* = 0.00099, *p* = 0.0025, respectively), entorhinal cortex (*p* = 0.00008), somatosensory cortex (*p* = 0.00008), and frontal cortex (*p* = 0.011); the FDR adjustment produced *p* < 0.05 for all structures. *Tgfb1* upregulation was still found in the ventral hippocampus 5 months after SE (*p* = 0.034, MU test); however, as was the case with *Tnf*, the FDR-adjusted *p* value was 0.21.

Notably, unlike in the components of the plasminogen system, there were no evident regional differences in the expression of *Tnf* and *Tgfb1*. Upregulation of *Tgfb1* in the latent period and upregulation of *Tnf* in the chronic period after SE was found both in the limbic structures and in the neocortex.

**Figure 5:**
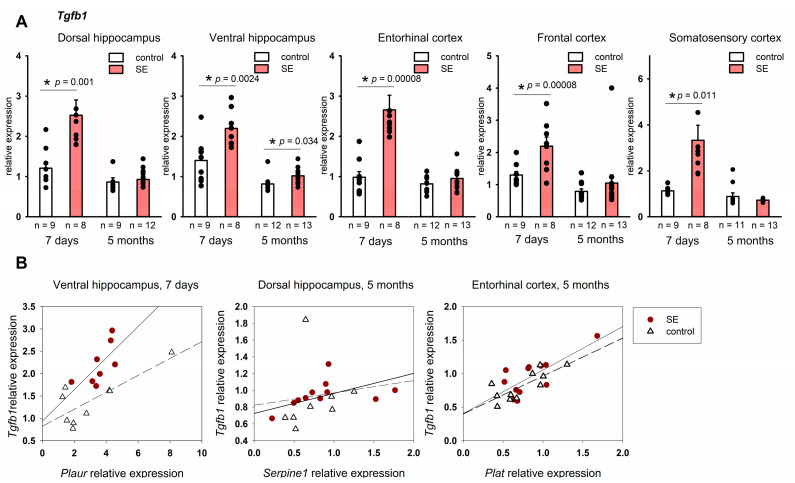
(**A**) Expression of *Tgfb1* in the hippocampus and cortex 7 days and 5 months after SE. Control—age-matched control group; SE—post SE group. *—statistically significant difference at *p* < 0.05; *n*—the number of samples in each group; raw *p*-values are shown, MU test. Data is presented as mean ± SEM. (**B**) correlations between *Plat* and *Tgfb1* expression in rats 7 days after SE (Spearman coefficient 0.74, *p* = 0.036) and in the control group (Spearman coefficient 0.35, *p* = 0.35) correlations between *Serpine1* and *Tgfb1* expression in rats 5 months after SE (Spearman coefficient 0.71, *p* = 0.013) and in the control group (Spearman coefficient 0.62, *p* = 0.102); correlations between *Plat* and *Tgfb1* expression in rats 5 months after SE (Spearman coefficient 0.59, *p* = 0.041) and in the control group (Spearman coefficient 0.64, *p* = 0.032).

The expression of *Tgfb1* in the ventral hippocampus of rats 7 days after SE correlated with *Plaur* expression (Spearman coefficient 0.74, *p* = 0.036), but the same effect was not observed in the control group. Five months after SE, statistically significant correlations were found between *Tgfb1* and *Serpine1* in the hippocampus in the post-SE group and *Tgfb1* and *Plat* in the entorhinal cortex of both control and post-SE rats (Figure 5B).

### 3.4. Sucrose Preference Test

**Figure 6:**
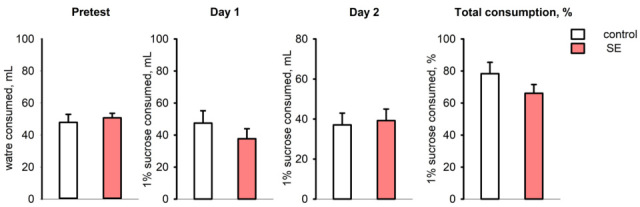
The volume of water consumed during the pre-test, 1% sucrose solution on test days 1 and 2, and total sucrose consumption per 48 h (% of total consumed liquid) in the sucrose preference test in rats 5 months after SE (*n* = 13) and in age-matched controls (*n* = 11).

**Figure 7:**
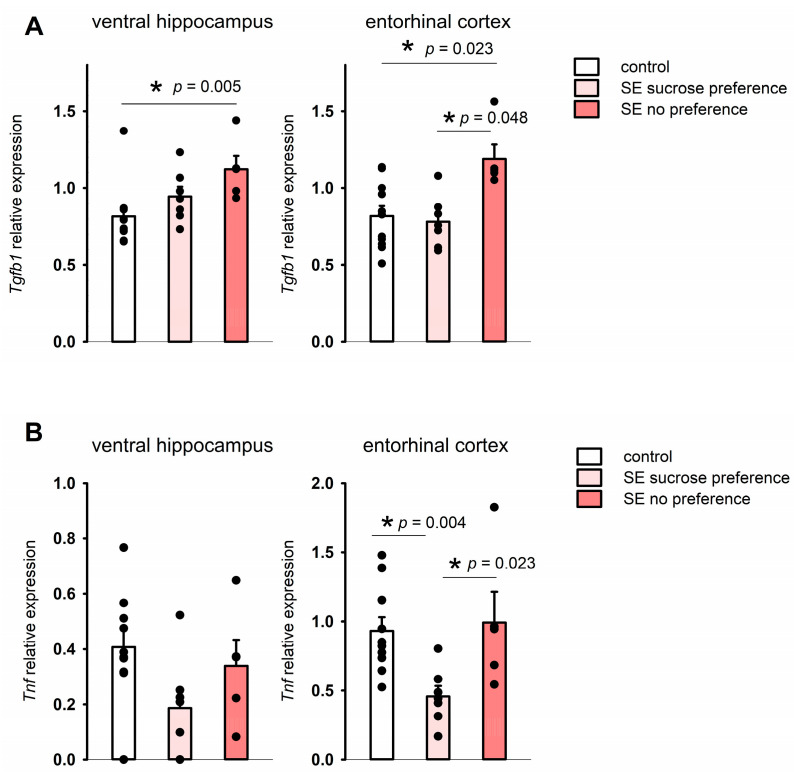
Expression of *Tgfb1* (**A**) and *Tnf* (**B**) in rats exhibiting anhedonia (SE no sucrose preference, *n* = 5), rats post-SE without anhedonia (*n* = 8), and no SE controls (*n* = 11) in the sucrose preference test 5 months after SE. *—statistically significant difference at *p* < 0.05; raw *p*-values are shown, MU test. Data is presented as mean ± SEM.

In the sucrose preference test, the control and post-SE groups did not show any differences in water consumption during the pre-test; the mean volume of consumed 1% sucrose also was not significantly different on both test days (Figure 6). The ratio of total sucrose solution consumption per 48 h was 78.35 ± 6.81% in the control group and 66.12 ± 5.45% in the post-SE group, so post-SE rats tended to have lower sucrose consumption, but it was not statistically significant (Figure 6). Evaluation of individual values of sucrose consumption showed that 8 out of 11 age-matched control rats exhibited a high consumption rate (over 80%) of 1% sucrose; at the same time, 5 out of 13 rats in the post-SE group showed no preference for 1% sucrose with a consumption rate of less than 60%. Therefore, the post-SE group was divided into two distinct subgroups depending on the presence (*n* = 8) or absence (*n* = 5) of sucrose preference. We compared the expression of the studied genes in the hippocampus and cortex of these subgroups and the control group (Figure 7). An increase in *Tgfb1* expression in the ventral hippocampus was more prominent in the rats demonstrating no sucrose preference in comparison to control rats (*p* = 0.009, Kruskal–Wallis test, *p* = 0.005, post hoc MU test). In the entorhinal cortex, *Tgfb1* expression was higher in the rats with no sucrose preference compared to rats with sucrose preference and the age-matched control group without SE (*p* = 0.049, Kruskal–Wallis test, *p* = 0.048 and *p* = 0.023 post hoc MU test) (Figure 7A). Interestingly, *Tnf* expression tended to decrease in the ventral hippocampus (*p* = 0.09, Kruskal–Wallis test, *p* = 0.035 post hoc MU test) and was reduced in the entorhinal cortex of post-SE rats showing sucrose preference compared to rats without SE (*p* = 0.007, Kruskal–Wallis test, *p* = 0.004, post hoc MU test), while rats exhibiting anhedonia had higher expression of *Tnf* that did not differ from the control group (Figure 7B). No difference in the expression of plasminogen activation system components was found between rats with the presence and absence of sucrose preference.

## 4. Discussion

Our findings show that SE induces region-specific, long-lasting dysregulation of the plasminogen activation system, with the entorhinal cortex being particularly susceptible to long-term alterations. Significant changes in the studied genes expression were observed 7 days after SE. In adult rats in lithium–pilocarpine SE, this time point is typically within the silent seizure-free period [51,57], while the acute effects of prolonged seizures, such as brain tissues edema, are already resolved [58,59]. It has been established that during this latent period preceding the development of chronic epilepsy, a number of processes takes place, including neuronal cell death, neuroinflammation, neurogenesis, gliogenesis, and plasticity [47]. In our study, we observed an increase in the expression of *Plat* encoding tPA in the hippocampus and *Plaur* encoding uPAR in the entorhinal cortex. This could be evidence of potential involvement of the plasminogen activation system in these pathological and reparative processes. tPA upregulation was reported to play a part in neurodegeneration, brain–blood barrier permeability, and neuroinflammation [6,60]. tPA upregulation in the hippocampus in the latent period after SE could be related to these processes and could indicate plastic changes triggered by SE [4]. The plasminogen activation system is known to respond rapidly to brain injury, including ischemia, trauma, and seizures [1,7,8]. An increase in *Plat* and *Plaur* expression is observed within hours after the impact on the brain [8,61]. An increase in *Plat* expression was reported in the hippocampus and temporal lobe in the latent period of post-SE epileptognesis [11,12,13], while in the chronic period it was found only in the hippocampal CA3 [12]. Our results also demonstrated *Plat* upregulation in the hippocampus in the latent period after SE, though this upregulation was not very prominent and was not maintained into the chronic phase. SE-induced *Plaur* upregulation in the hippocampus was reported 24–48 h after seizures, but returned to baseline values by day 4 [10]. Our results extend these findings by showing that while there was no significant *Plaur* upregulation in the hippocampus 7 days after SE, which is consistent with previous findings [10], a sustained *Plaur* elevation was observed in the entorhinal cortex. Moreover, *Plaur* upregulation in the entorhinal cortex persisted even 5 months after SE. uPAR signaling is known to be involved in regulating cell survival and proliferation [62]; uPAR binding with urokinase-type plasminogen activator uPA participates in the regulation of axonal growth and neurorepair, and it also is involved in the regulation of synaptic plasticity [63,64]. At the same time, uPAR deficiency was shown to increase seizure susceptibility [65]. Upregulation of uPAR is also associated with microglia activation and neuroinflammation [66]. Persistent *Plaur* upregulation in our study could reflect long-term plasticity changes and chronic neuroinflammation in the entorhinal cortex triggered by initial damage after SE; however, it is difficult to assess the input of recurrent epileptic activity due to low incidence of spontaneous seizures in our rats.

Although the expression of *Serpine1* in the brain tissue is low under normal conditions, and we observed no significant changes in *Serpine1* and *Plat* expression during the chronic period following SE, our findings reveal positive correlations between expression of *Serpine1*, *Plat*, and *Plaur* in the rat hippocampus and cortex. Interestingly, these associations were disrupted after SE, as no significant correlations were detected in the hippocampus or cortex of post-SE rats. Notably, in the entorhinal cortex, this disruption persisted for as long as five months after SE. Our data do not allow us to establish a causal relationship between the expression of PA system genes, since it is regulated by a large number of factors, and correlation coefficients do not necessarily reflect direct interactions between genes. However, together with long-term alterations in the mRNA expression of *Plaur* observed in the entorhinal cortex, it is possible to suggest lasting transcriptional dysregulation within the plasminogen activation system induced by SE. This sustained disruption may contribute to the pathological remodeling of neuronal circuits and could play a role in the chronic consequences of SE.

Regional specificity of the changes in PA system gene expression generally corresponded to the pattern of brain damage observed after SE, with the most prominent changes in the hippocampus and entorhinal cortex [51,58]. Nevertheless, despite the lack of significant changes in the expression of PA system components in the neocortical regions, our results still suggest the possible disruption of the PA system in the frontal cortex as well. At the same time, there were changes in inflammatory cytokine expression in the neocortical regions in the latent period after SE.

The absence of a significant increase in pro-inflammatory cytokines expression in the hippocampus 7 days after SE is in agreement with previous studies. Rapid upregulation of *Tnf* and *Il1b* in the hippocampus within hours after the initiation of seizure activity was reported in various models of seizures, including SE models [16,17], and by day 7 after seizures, the mRNA expression in the hippocampus returned to control levels. Our results show no increase in *Tnf* and *Il1b* expression in the hippocampus 7 days after SE. However, in the neocortex, *Il1b* was still upregulated 7 days after SE, indicating the ongoing neuroinflammatory processes during this time period. This shows that the dynamics of neuroinflammatory processes in the cortex are different, probably delayed or extended, in comparison to the hippocampus.

One of the key roles of the plasminogen activation system in the brain has been reported to be the regulation of neuroinflammation [67]. We have found positive correlations between the mRNA expression of inflammatory cytokines and components of the plasminogen activation system, suggesting a potential association between upregulated tPA mRNA expression and neuroinflammation following SE. These findings align with the role of tPA in neuroinflammation. As a known activator of microglia [67,68], tPA can initiate the release of a number of pro-inflammatory cytokines, including IL-1β, by activated microglia, so positive correlations between *Plat* and *Il1b* expression in the frontal cortex with elevated *Il1b* expression in the latent period after SE may reflect this process at the transcriptional level.

Interestingly, while no upregulation of *Tnf* in the hippocampus and cortex of rats was observed 7 days after SE, 5 months after SE *Tnf* expression was reduced in comparison with controls. In rodents, seizures were shown to induce TNF-α upregulation in astrocytes [17], endothelial cells, and microglia [69]. TNF-α can also be expressed in neurons [70]. We observed a tendency toward *Tnf* downregulation 5 months after SE in comparison with no-SE controls. This could have occurred due to a decrease in *Tnf* expression in glia or the loss of subpopulations of cells expressing *Tnf.*

Various damaging events and pathologic conditions such as acute stroke and a number of neurodegenerative disorders are accompanied by an increase in TGF-β signaling [24,71,72]. In the pilocarpine model of seizures, an increase in TGF-β1 protein level in the hippocampus was observed during the first week after SE [73], and an increase in TGF-β signaling has been reported to be involved in epileptogenesis in animal models [34,35]. Our findings demonstrated that SE induced *Tgfb1* upregulation in both limbic structures and the neocortex, which persisted for at least 7 days post-SE, and in the hippocampus, an increase in its expression was observed very late in the chronic period after SE. It was reported that prolonged overexpression of TGF-β1 can induce structural changes in the hippocampus, leading to impairments in learning [74]. Importantly, such cognitive impairments are a characteristic feature of post-SE epilepsy models, including the lithium–pilocarpine model [75,76,77]. The persistent upregulation of *Tgfb1* we observed may be one of the mechanisms contributing to the development of these impairments.

TGF-β was reported to induce upregulation of PAI-1 at the transcriptional level [78,79]. Our results showed positive correlations between *Tgfb1* expression and expression of the components of the plasminogen activation system in the rat brain. This association between *Tgfb1* and plasminogen activation system components expression could indicate a potential involvement of *Tgfb1* in the regulation of plasminogen activation system at the transcriptional level. An increase in *Tgfb1* expression together with the plasminogen activation system upregulation in our study might also be evidence of the involvement of TGF-β1 in the pro-inflammatory response triggered by the prolonged seizures during SE.

The inability to experience pleasure from normally pleasurable activities, referred to as anhedonia, is one of the key symptoms of depressive disorders [80]. In rodents, anhedonia is typically determined as lack of preference for sweet taste [81,82]. In the sucrose preference test for depressive-like behavior, a decrease in consumption of sweetened solution to below 60–65% is typically considered as a sign of anhedonia [83]. Rodent models of seizures, including the lithium–pilocarpine model, are characterized by behavioral and biochemical alterations typical for depression, and inflammatory cytokines are reported to be involved in these processes [49,84]. We have observed upregulation of *Tgfb1* in two brain regions—the ventral hippocampus and entorhinal cortex—of rats exhibiting depressive-like behavior in the chronic period after SE. In addition, rats with depressive-like behavior had higher *Tnf* expression in the same regions as *Tgfb1* in comparison to rats that were not exhibiting anhedonia, but it was not different from the control animals. In the present study, we could not assess the contribution of spontaneous epileptic activity to the changes in mRNA expression due to low incidence of spontaneous seizures. It is possible that animals with more prominent seizure activity have a higher probability of developing depressive-like behavior. Therefore, there is a possibility that an increase in *Tgfb1* expression was related to epileptogenesis in rats with anhedonia. However, this could be a result of long-term post-SE changes that were not directly related to the development of spontaneous seizures but could affect the behavioral outcome. We should also note that the difference was found between subgroups of rats that survived 5 months after SE, hence the number of animals per subgroup was small. This makes it difficult to evaluate the input of each factor to the post-SE outcome. Further studies on larger group of animals could provide more insight into the relationship between *Tgfb1* and *Tnf* expression, seizures, and the development of depressive-like behavior.

The association between inflammatory cytokines and the development of depression was shown both in animal models and in patients with depressive disorders [36,85]. In animals, pro-inflammatory cytokines induce a complex of behavioral and physiological changes, including changes in food intake and anhedonia [86], and TNF-α upregulation is associated with depressive disorders and depressive-like behavior in animal models [87]. TGF-β1 upregulation accompanied the depressive-like behavior induced by lipopolysaccharide administration [88]. Our findings are in line with previous studies and support the possible role of TNF-α and TGF-β1 in the development of depressive-like behavior in post-SE rats.

## 5. Conclusions

To summarize our findings, we have shown that SE triggers long-term region-specific dysregulation of the plasminogen activation system at the transcriptional level, which persists for months after SE. Changes in the expression of plasminogen activation system components are accompanied by alterations in the expression of pro-inflammatory cytokine *Il1b* and growth factor *Tgfb1*. Higher levels of *Tgfb1* expression, in addition to correlation with higher levels of plasminogen activation system expression, were observed in rats with depressive-like behavior in the chronic period after SE, which could be evidence of the possible role of TGF-β1 in the development of post-SE behavioral impairments. The development of depressive-like behavior in rats in the chronic period after SE was accompanied by higher expression of *Tgfb1* and *Tnf* in the hippocampus and entorhinal cortex.

## Figures and Tables

**Table 1:** The sequences of primers used for qPCR.

Gene	Forward Primer	Reverse Primer	Amplicon Size
*Tgfb1*	GCGCCTGCAGAGATTCAAGTCAAC	TCAGGCGTATCAGTGGGGGTCA	117
*Tnf*	GTCCAACTCCGGGCTCAGAAT	ACTCCCCCGATCCACTCAG	173
*Il1b*	TCTGTGACTCGTGGGATGAT	CACTTGTTGGCTTATGTTCTGTC	161
*Plat*	CAGTGCCCTGACGGATTTGTTG	AGGGCTTCACGTCTCGGTCT	241
*Plaur*	CGGGCACAGCAGGTTTCCATAG	CTCCGGTTTCCCAGCACATCTAAG	244
*Serpine1*	TCGGCACAATCCAACAGAGACA	CCAGTGCCGGGGTAAGAAAGA	184
*Ywhaz*	TTGAGCAGAAGACGGAAGGT	GAAGCATTGGGGATCAAGAA	136
*Hprt*	CGTCGTGATTAGTGATGATGAAC	CAAGTCTTTCAGTCCTGTCCATAA	128
*Osbp*	TCCGGGAGACTTTACCTTCACTT	GTGTCACCCTCTTATCAACCACC	100

## Data Availability

The original data presented in the study are openly available at [https://doi.org/10.6084/m9.figshare.30071905/https://figshare.com/s/d6c03484049243917b50 (accessed on 30 September 2025)].

## Abbreviations

The following abbreviations are used in this manuscript:

DNase	Deoxyribonuclease
IL-1β	Interleukin-1 beta (gene: *Il1b*)
PA	Plasminogen Activation
PAI-1	Plasminogen Activator Inhibitor-1 (gene: *Serpine1*)
qPCR	Quantitative Polymerase Chain Reaction
SE	Status Epilepticus
SEM	Standard Error of the Mean
TGF-β1	Transforming Growth Factor beta 1 (gene: *Tgfb1*)
TLE	Temporal Lobe Epilepsy
TNF-α	Tumor Necrosis Factor alpha (gene: *Tnf*)
tPA	Tissue Plasminogen Activator (gene: *Plat*)
uPAR	Urokinase-type Plasminogen Activator Receptor (gene: *Plaur*)
